# Intravenous Arginine Administration Benefits CD4^+^ T-Cell Homeostasis and Attenuates Liver Inflammation in Mice with Polymicrobial Sepsis

**DOI:** 10.3390/nu12041047

**Published:** 2020-04-10

**Authors:** Chiu-Li Yeh, Sharon Angela Tanuseputero, Jin-Ming Wu, Yi-Ru Tseng, Po-Jen Yang, Po-Chu Lee, Sung-Ling Yeh, Ming-Tsan Lin

**Affiliations:** 1 School of Nutrition and Health Sciences, College of Nutrition, Taipei Medical University, Taipei 11031, Taiwan; clyeh@tmu.edu.tw (C.-L.Y.); sharonangela93@gmail.com (S.A.T.); b506104054@tmu.edu.tw (Y.-R.T.); sangling@tmu.edu.tw (S.-L.Y.); 2 Nutrition Research Center, Taipei Medical University Hospital, Taipei 11031, Taiwan; 3 Research Center of Geriatric Nutrition, College of Nutrition, Taipei Medical University, Taipei 11031, Taiwan; 4 Department of Surgery, National Taiwan University Hospital and College of Medicine, National Taiwan University, Taipei 10002, Taiwan; wujm0531@ntu.edu.tw (J.-M.W.); paulpjyang@gmail.com (P.-J.Y.); d97421103@ntu.edu.tw (P.-C.L.)

**Keywords:** arginine, sepsis, nitric oxide, T helper cell, regulatory T cell, liver inflammation

## Abstract

This study investigated the effects of a single dose of arginine (Arg) administration at the beginning of sepsis on CD4^+^ T-cell regulation and liver inflammation in C57BL/6J mice. Mice were divided into normal control (NC), sham (SH), sepsis saline (SS), and sepsis Arg (SA) groups. An inducible nitric oxide (NO) synthase (iNOS) inhibitor was administered to additional sepsis groups to evaluate the role of NO during sepsis. Sepsis was induced using cecal ligation and puncture (CLP). The SS and SA groups received saline or Arg (300 mg/kg body weight) via tail vein 1 h after CLP. Mice were euthanized at 12 and 24 h post-CLP. Blood, para-aortic lymph nodes, and liver tissues were collected for further measurement. The findings showed that sepsis resulted in decreases in blood and para-aortic lymph node CD4^+^ T-cell percentages, whereas percentages of interleukin (IL)-4- and IL-17-expressing CD4^+^ T cells were upregulated. Compared to the SS group, Arg administration resulted in maintained circulating and para-aortic lymph node CD4^+^ T cells, an increased Th1/Th2 ratio, and a reduced Th17/Treg ratio post-CLP. In addition, levels of plasma liver injury markers and expression of inflammatory genes in liver decreased. These results suggest that a single dose of Arg administered after CLP increased Arg availability, sustained CD4^+^ T-cell populations, elicited more-balanced Th1/Th2/Th17/Treg polarization in the circulation and the para-aortic lymph nodes, and attenuated liver inflammation in sepsis. The favorable effects of Arg were abrogated when an iNOS inhibitor was administered, which indicated that NO may be participated in regulating the homeostasis of Th/Treg cells and subsequent liver inflammation during sepsis.

## 1. Introduction

Sepsis is defined as a life-threatening syndrome with multiorgan dysfunction which is caused by dysregulation of the host immune response to infection [1]. Severe infection and inflammation during sepsis activate innate and adaptive immunity in the body. Invading pathogens exaggerate the production of inflammatory mediators. To regulate this process and protect against excessive damage, anti-inflammatory mediators are subsequently produced that may result in compensatory anti-inflammatory response syndrome [2]. Both pro- and anti-inflammatory reactions concurrently occur during sepsis. The imbalance between these two reactions leads to the dysregulation of inflammatory response and consequently results in irreversible multiorgan injury [3]. Restoring the homeostasis of pro- and anti-inflammatory reactions may balance systemic immune responses, reduce sepsis-induced comorbidity, and protect organs from damage [4].

Disease-associated T lymphocyte dysfunction and dysregulation aggravate the inflammatory response and subsequent outcomes [5]. CD4^+^ T cells are an important lymphocyte subset that influences cellular and humoral immunity [6]. Dysfunction of CD4^+^ T-cell and imbalance of T helper (Th) and regulatory T (Treg) populations are characteristics of sepsis [7]. CD4^+^ T cells can be divided into several subsets that are differentiated to different effectors which produce distinct cytokines [5]. Th1 cells secrete interferon (IFN)-*γ* and interleukin (IL)-2 to enhance cellular immunity [8]. Th2 cells produce IL-4, IL-5, and IL-13 to promote humoral immunity [9,10]. IL-17 and IL-22, which are secreted by Th17 cells, play crucial roles in recruiting neutrophils to injury sites [7]. Treg cells are a different subset of T-cells with actions against Th17 [11]. Excessive Treg proliferation, CD4^+^ T-cell depletion, and alterations of CD4^+^ T-cell subset distributions and effector functions may contribute to higher risks of secondary infection [5,12]. Restoration of the balance in Th1/Th2/Th17/Treg responses can affect disease outcomes and is important in treating sepsis.

In healthy subjects, arginine (Arg) is a non-essential amino acid that serves as the precursor of various physiological components and metabolites [13]. However, it may become essential in metabolically stressed conditions as the Arg supply is far less than required by the physiological demands of the body during such situations [14]. Sepsis is an Arg-deficient state. In sepsis, the availability of Arg is limited because de novo synthesis is impaired and catabolism is enhanced [15]. Animal studies reported that supplemental Arg promotes wound healing, protects against bacterial infections, enhances lymphocyte proliferation, and improves survival in sepsis [16,17,18,19]. However, there are controversies regarding Arg supplementation during sepsis and in critically ill patients [20,21,22,23]. The optimal administered dosage, route, and intervention schedule in sepsis patients are still being investigated [24].

Arg is the co-substrate of arginase and nitric oxide synthase (NOS). Arginase converts Arg to urea and ornithine while nitric oxide (NO) and citrulline are produced via NOS. There are 3 isoforms of NOS: neuronal NOS (nNOS), inducible NOS (iNOS), and endothelial NOS (eNOS). The total NO production depends on the activities of the NOS enzymes [15]. NO is a signal molecule with multiple functions in both healthy and diseased states. Earlier work implied that excessive NO production was associated with the occurrence of septic shock; however, general inhibition of NOS increases mortality [25]. Those findings suggest that appropriate NO production is important in regulating metabolic homeostasis and immune responses during sepsis. Because the activities of constitutive NOS decreased during sepsis [15], some investigators suggest NO production should be encouraged with the use of supplemental Arg [26]. In this study, we intravenously administered 300 mg Arg/kg body weight (BW) after sepsis to investigate the influence of Arg on CD4^+^ T-cell regulation during sepsis. This dosage of Arg has been demonstrated to reduce systemic inflammation and maintain vascular homeostasis in sepsis [19]. Inducible NOS-derived NO is produced in response to inflammatory reaction. In order to understand the roles of iNO in regulating sepsis-associated T-cell dysregulation, an iNOS inhibitor was used. Since the liver is one of the target organs that is frequently affected and liver dysfunction often occurs in the early stage of sepsis [4], the influence of Arg administration on liver injury was evaluated. We used a cecal ligation and puncture (CLP) mouse model in this study because CLP mimics pathophysiological changes of human septic patients [27] and is the most frequently used rodent model in sepsis research.

## 2. Materials and Methods

### 2.1. Animals

Male C57BL/6J mice (aged ~5–6 weeks and ~20–22 g in weight) were used in this experiment. All mice were allowed to acclimatize in room with controlled temperature (21 ± 2 °C) and humidity (~50–55%) with a 12 h light–dark cycle at the Laboratory Animal Center of Taipei Medical University for 2 weeks before the study. During the study period, all mice were given standard rodent chow and water ad libitum. Care of laboratory animals and the experimental protocols were all approved by Animal Care and Use Committee of Taipei Medical University (LAC-2017-0421).

### 2.2. Study Protocol

**Figure 1:**
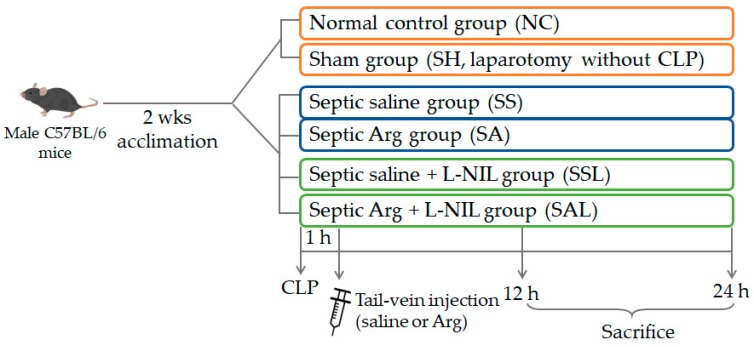
The illustration of experimental grouping and protocol. Arg, arginine; CLP, cecal ligation and puncture; L-NIL, L-N(6)-iminoethyl-lysine, an inhibitor of inducible nitric oxide synthase.

Mice were randomly assigned to four groups: normal control group (NC, *n* = 8), sham group (SH, *n* = 8), sepsis saline group (SS, *n* = 20), and sepsis Arg group (SA, *n* = 20). CLP operation was performed to induce polymicrobial peritonitis sepsis as previously described [27]. Mice in the sepsis groups were intraperitoneally injected with Zoletil (Virbac, Carros, France; 25 mg/kg body weight (BW)) and Rompun (Bayer, Leverkusen, Germany; 10 mg/kg BW) for anesthetization. An ~1–1.5 cm incision was made into the abdominal wall with subsequent opening of the peritoneum. The cecum was extracted and was ligated at approximately 50% below the ileocecal valve. The cecum was punctured through using a 23-gauge needle. A small amount of feces was squeezed out and smeared onto the abdomen. The incision was then closed with a continuous suturing technique. To ensure consistency, all CLP operations were standardized and carried out by the same operator. Animals were rehydrated with sterile saline (40 mL/kg BW) subcutaneously after the operation. Mice in the SH group were subjected to laparotomy without CLP and sacrificed 1 h post-operation. After surgery, all mice were allowed free access to rodent chow and water. Postoperative pain was managed by treatment with 100 μL of 0.25% bupivacaine at the incision site before skin closure. One hour after CLP, mice were intravenously injected with one bolus (100 µL) of either saline or Arg (300 mg/kg BW) via a tail vein. According to the schedule, mice were sacrificed at 12 or 24 h after CLP. Mice were euthanized by cardiac puncture under ether anesthesia. Blood samples were collected and para-aortic lymph nodes and liver tissues were excised. Fresh whole blood was used to measure the population of CD4^+^ T-cell subtypes. Part of the blood sample was centrifuged at 700*g* and 4 °C for 15 min to obtain plasma which was stored in −80 °C for further measurements. To investigate the role of iNO, L-N(6)-iminoethyl-lysine (L-NIL) (Sigma, St. Louis, MO, USA), a specific iNOS inhibitor, was administered to additional sepsis groups of septic saline with L-NIL (SSL, *n* = 16) and septic Arg with L-NIL (SAL, *n* = 16). L-NIL (3 mg/kg BW) was delivered intraperitoneally at the end of CLP and 6 h after sepsis induction [28]. Mice in the SSL and SAL groups were sacrificed at 12 or 24 h post-CLP to collect blood, para-aortic lymph nodes, and liver tissues. The experimental grouping and protocol is shown in Figure 1.

### 2.3. Measurements of Plasma Biochemical Parameters

Concentrations of alanine transaminase (ALT), aspartate transaminase (AST), and galectin-3 were analyzed by commercial kits (Thermo Fisher Scientific, Waltham, MA, USA). All procedures followed the instructions provided by manufacturer.

### 2.4. Evaluation of Plasma Amino Acid Concentrations and Arg Availability

Plasma samples were prepared using a Waters AccQTag derivatization kit (Manchester, UK) and subjected to ultra-performance liquid chromatography (UPLC) separation using the ACQUITY UPLC system (Waters). A multiple reaction monitoring (MRM) analysis was performed using a Xevo TQ-XS (Waters) mass spectrometer. Data were analyzed using Waters MassLynx 4.2 software and quantified using TargetLynx. Arginine availability index (AAI) was used to evaluate the relative availability of Arg and was calculated as [Arg]/([ornithine] + [lysine]) [29].

### 2.5. Measurements of CD4^+^ T-Cell Subpopulations

Whole blood and para-aortic lymph nodes were used for measuring Th1, Th2, Th17, and Treg populations. After incubation for 15 min, red blood cells (RBCs) were lysed using RBC Lysis Buffer (BioLegend, San Diego, CA, USA). Lymph nodes were homogenized and washed in Dulbecco’s modified Eagle medium (DMEM; Gibco, Waltham, MA, USA) and staining buffer (2% bovine serum albumin in phosphate-buffered saline), followed by resuspension in staining buffer. Intracellular staining was performed using Intracellular Staining Permeabilization Wash Buffer and Fixation Buffer (BioLegend) for Th cells and Foxp3/Transcription Factor Staining Buffer (Invitrogen, Carlsbad, CA, USA) for Treg cells. Flow cytometric analysis was conducted according to standard settings on a BD FACS-Canto II flow cytometer (BD Biosciences, San Diego, CA, USA), and data were analyzed with BD FACSDiva^TM^ Software v. 8.0 (BD Biosciences). Lymph node suspensions (100 µL) and whole blood (50 µL) were stained according to the instruction manual. The following antibodies were used for intracellular cytokine staining: pacific blue-anti-CD3 (17A2, BioLegend) and PerCP-anti-CD4 (GK1.5, BioLegend), allophycocyanin (APC)-anti-IFN-γ (XMG1.2, BioLegend), phycoerythrin (PE)-anti-IL4 (11B11, BioLegend), and fluorescein isothiocyanate (FITC)-anti-IL17A (TC11-18H10.1, BioLegend). Populations of Treg cells were analyzed by intracellular staining using APC-anti-CD4 (GK1.5, BioLegend), PE anti-CD25 (3C7, BioLegend), and FITC-Foxp3 antibodies (150D, BioLegend). During data analysis, 10,000 events which were considered to be from the lymphocyte population were gated for Th1 (CD3^+^/CD4^+^/IFN-γ^+^), Th2 (CD3^+^/CD4^+^/IL-4^+^), Th17 (CD3^+^/CD4^+^/IL-17A^+^), and Treg cells (CD4^+^/CD25^+^/FoxP3^+^).

### 2.6. Measurements of Thiobarbituric Acid-Reactive Substances (TBARSs) in Liver Tissues

Using a homogenizer, liver tissues were prepared in 0.01 M phosphate buffer (pH 7.4) with 1.15% KCl at 4 °C to create a 15% homogenate. Homogenates were centrifuged and the supernatants were obtained for the analysis of TBARS. Protein concentrations of supernatants were analyzed by Lowry’s method. TBARSs are formed as a byproduct of lipid peroxidation. Malondialdehyde (MDA) is one of the end products of lipid peroxidation. Assay of TBARS measures MDA in the sample, which was determined by a previously described method [30].

### 2.7. RNA Extraction and Quantitative Real-Time Polymerase Chain Reaction (qPCR) of Liver Tissues

Total RNA was extracted from liver tissues by TRIzol reagent (Invitrogen, Carlsbad, CA, USA). According to the standard protocols, 2.5 μg RNA was reverse-transcribed using a RevertAid First Strand cDNA Synthesis Kit (Thermo Scientific, Waltham, MA, USA) with oligo (dT)_18_ primers. qPCR was performed in 96-well plates on an ABI 7300 Real-Time PCR System (Applied Biosystems, Foster City, CA, USA). The primers used are presented in Table 1. The respective gene expression was analyzed in a total volume of 25 μL containing 1× Maxima SYBR Green/ROX qPCR Master Mix (Thermo Scientific), 200 nM of each primer and complementary (c)DNA 50 ng. The PCR running conditions were one cycle at 95 °C for 10 min and 40 cycles of amplification at 95 °C for 15 s, 60 °C for 1 min. Subsequently, a dissociation program was applied with one cycle at 95 °C for 15 s, 60 °C for 1 min, and 95 °C for 15 s. Dissociation curve (DC) analysis was recommended by the PCR system. To confirm the specificity of the qPCR, no-template controls and a melting curve analysis were used in this study. The multiple changes of messenger (m)RNA were calculated using the equation 2^−△△Ct^.

### 2.8. Statistical Analysis

All analyzed study groups were initially tested for normality using the Kolmogorov–Smirnov procedure with Lilliefors correction and all groups fulfilled the criteria of normal distribution. Data are presented as the means ± standard deviation (SD). Results were analyzed using GraphPad Prism 5 software (La Jolla, CA, USA). Differences among groups were evaluated using a one-way analysis of variance (ANOVA) followed by Tukey’s post hoc test. Values were considered statistically significant at *p* < 0.05.

## 3. Results

### 3.1. Plasma Biochemical Markers and Liver Lipid Peroxide Concentrations

Plasma ALT, AST, and galectin-3 concentrations in the sepsis groups at either 12 or 24 h post-CLP were significantly higher than those in the NC and SH groups. Compared to the SS group, the SA group had significantly lower ALT, AST, and galectin-3 levels at both time points post-CLP. The SS group had higher liver MDA concentrations than the NC and SH groups, while this phenomenon was not noted in the SA group. The sepsis groups with L-NIL administration also showed higher plasma ALT, AST, and galectin-3 levels than the control groups. However, these biochemical markers did not differ between the SSL and SAL groups at either time point. No differences in liver MDA concentrations were observed between the two sepsis groups with L-NIL after CLP (Table 2).

### 3.2. Plasma Amino Acid Concentrations and Arg Availability Assessment

Plasma Arg and citrulline levels were measured 24 h after CLP. The SS group had lower Arg and citrulline concentrations than those of the NC group. The SA group exhibited higher Arg and citrulline levels than the SS group and had no differences from the NC group (Arg (μM): NC 75.6 ± 13.7, SA 52.1 ± 6.5 vs. SS 25.8 ± 7.7, *p* < 0.05; citrulline (μM): NC 68.2 ± 14.6, SA 55.6 ± 3.2 vs. SS 32.1 ±1.9, *p* < 0.05). The relative Arg availability was assessed by AAI. The AAI values were significantly higher in the NC group than the sepsis groups (NC 0.268 ± 0.066 vs. SS 0.082 ± 0.023 and SA 0.176 ± 0.009, *p* < 0.05). Compared to the SS group, the SA group had higher AAI values at 24 h after CLP (*p* < 0.05).

### 3.3. CD4^+^ T Lymphocyte Populations in Blood

**Figure 2:**
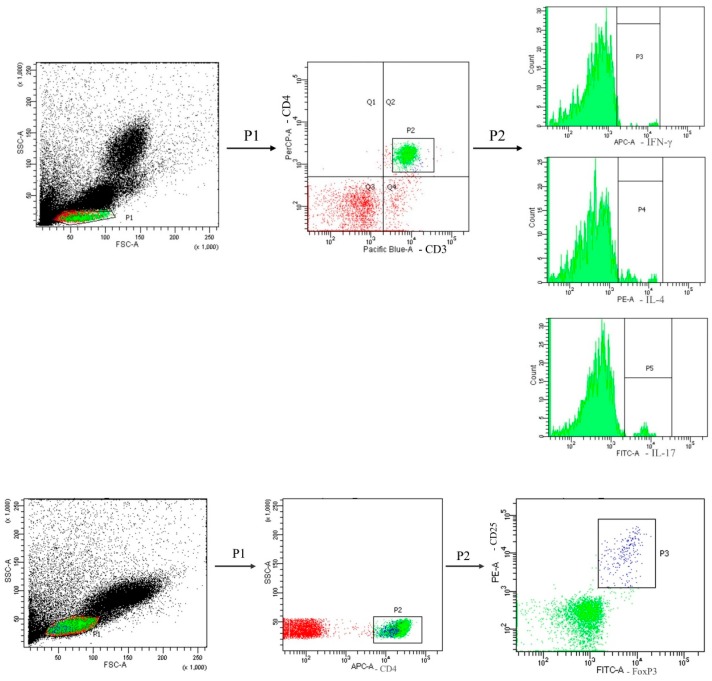
Representative flow cytometry data to illustrate the gating strategy for lymphocytes in blood. Lymphocytes were first identified based on low FSC and SSC characteristics. CD4-positive (CD4+) lymphocytes were gated to analyze the percentages of IFN-γ-, IL-4-, and IL-17-expressing CD4+ cells. Treg cells were identified as Foxp3^+^ CD25^+^ in CD4^+^ cells. Different cytokine-expressing CD4^+^ cells and Treg percentages among groups are shown in Figure 4 and Figure 6.

**Figure 3:**
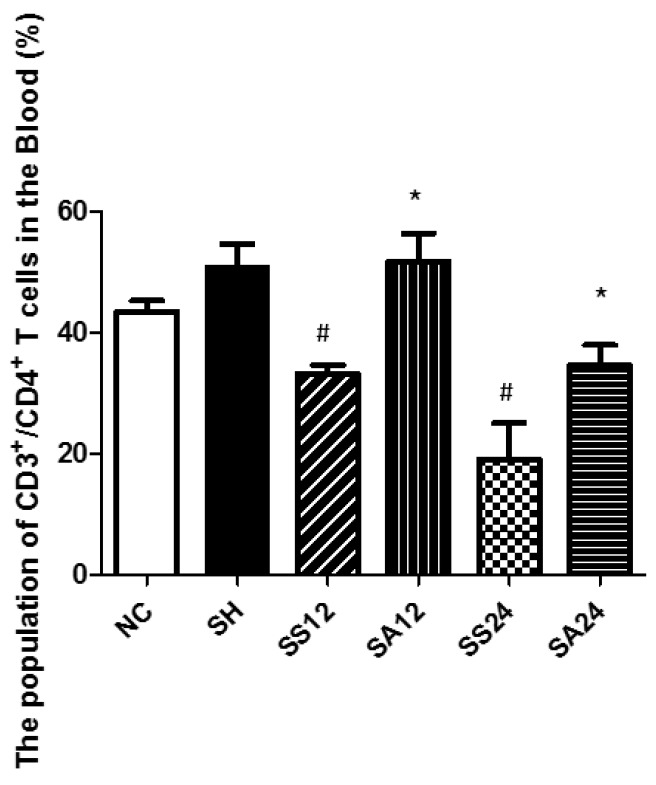
Percentages of CD4+ T helper (Th) cells in blood. NC, normal control group; SH, sham group; SS12, septic saline group sacrificed at 12 h after cecal ligation and puncture (CLP); SA12, septic arginine (Arg) group sacrificed at 12 h after CLP; SS24, septic saline group sacrificed at 24 h after CLP; SA24, septic Arg group sacrificed at 24 h after CLP. Data were analyzed using a one-way ANOVA with Tukey’s post hoc test and are presented as the mean ± SD. *n* = 8 for each group. ^#^ Significantly differs from the NC group (*p* < 0.05). * Significantly differs from the SS group at the same time point (*p* < 0.05).

**Figure 4:**
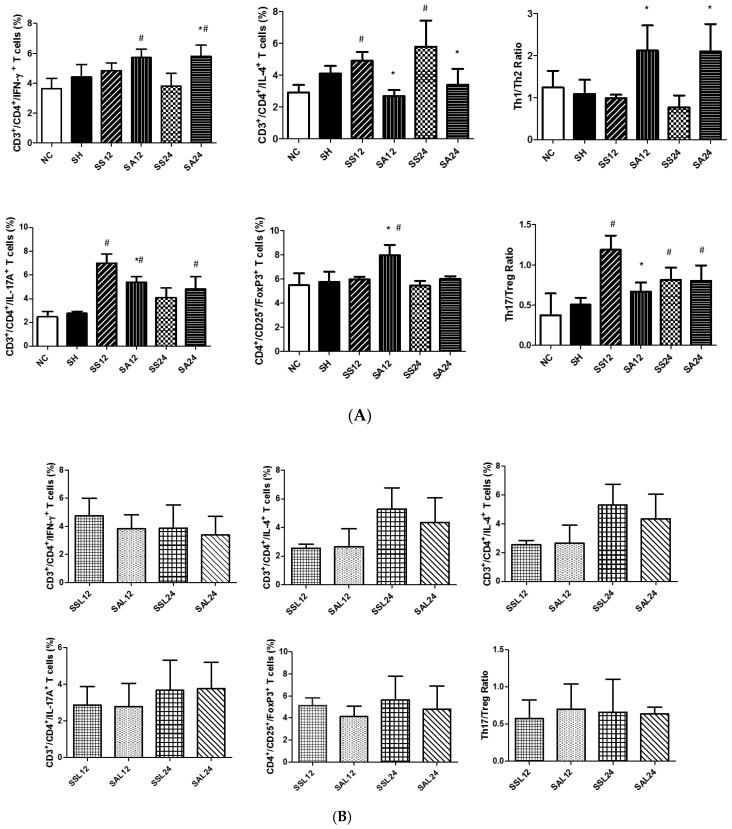
Blood percentages of (**A**) CD4+T-cell subsets including Th1, Th2, Th17, regulatory T (Treg) cell distributions, and the Th1/Th2 and Th17/Treg ratios. NC, normal control group; SH, sham group; SS12, septic saline group sacrificed at 12 h after cecal ligation and puncture (CLP); SA12, septic arginine (Arg) group sacrificed at 12 h after CLP; SS24, septic saline group sacrificed at 24 h after CLP; SA24, septic Arg group sacrificed at 24 h after CLP. (**B**) CD4+T-cell subsets and the Th1/Th2 and Th17/Treg ratios in sepsis groups with an inducible nitric oxide synthase (iNOS) inhibitor. SSL12, SS12 group with iNOS inhibitor; SAL12, SA12 with the iNOS inhibitor; SSL24, SS24 with the iNOS inhibitor; SAL24, SA24 with the iNOS inhibitor. Percentages of Th1 (interferon-γ-expressing cells), Th2 (interleukin-4-expressing cells), and Th17 cells (interleukin-17A-expressing cells) among CD4^+^ lymphocytes were measured. Treg cells are presented as a percentage of CD25^+^Foxp3^+^ cells among CD4^+^ lymphocytes. Data were analyzed using a one-way ANOVA with Tukey’s post hoc test and are presented as the mean ± SD. *n* = 8 for each group. ^#^ Significantly differs from the NC group (*p* < 0.05). * Significantly differs from the SS group at the same time point (*p* < 0.05).

A typical flow cytometry data was shown in Figure 2 to illustrate the gating strategy for lymphocytes in blood. CD4^+^ lymphocytes were gated to analyze the percentages of different cytokine-expressing CD4^+^ cells. Treg cells were identified as CD25^+^Foxp3^+^ in CD4^+^ cells. Sepsis led to decreased percentages of CD4^+^ lymphocytes (CD4^+^ in the gated CD3^+^ population) in blood. Sepsis groups with Arg maintained their percentages of CD4^+^ T cells and exhibited no difference from the NC group (Figure 3). Concerning subsets of CD4^+^ T cells, there were no differences in Th1, Treg percentages and the Th1/Th2 ratio between the SS and NC groups. However, percentages of Th2 and Th17 cells and the Th17/Treg ratio in the SS group were significantly higher than those in the NC group at 12 and/or 24 h post-CLP. Compared to the SS group, the SA group exhibited higher Th1, Treg, and Th1/Th2 ratio and lower Th2, Th17, and Th17/Treg ratio at 12 and/or 24 h after CLP (Figure 4A). In the sepsis groups treated with L-NIL, no differences in the Th or Treg cell subpopulation distributions were observed among the groups at different time points (Figure 4B).

### 3.4. Distribution of T Lymphocyte Subpopulations in Para-Aortic Lymph Nodes

**Figure 5:**
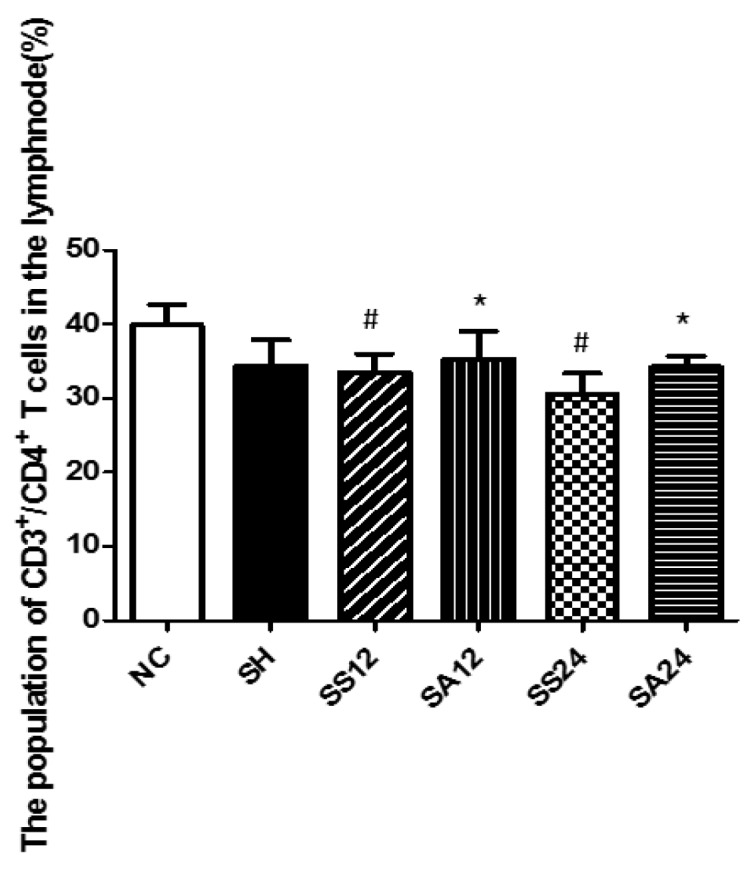
Percentages of CD4+ T helper (Th) cells in para-aortic lymph nodes. The description of the groups are found in the legend of Figure 2. Data were analyzed using a one-way ANOVA with Tukey’s post hoc test and are presented as the mean ± SD. *n* = 8 for each group. ^#^ Significantly differs from the NC group (*p* < 0.05). * Significantly differs from the SS group at the same time point (*p* < 0.05).

**Figure 6:**
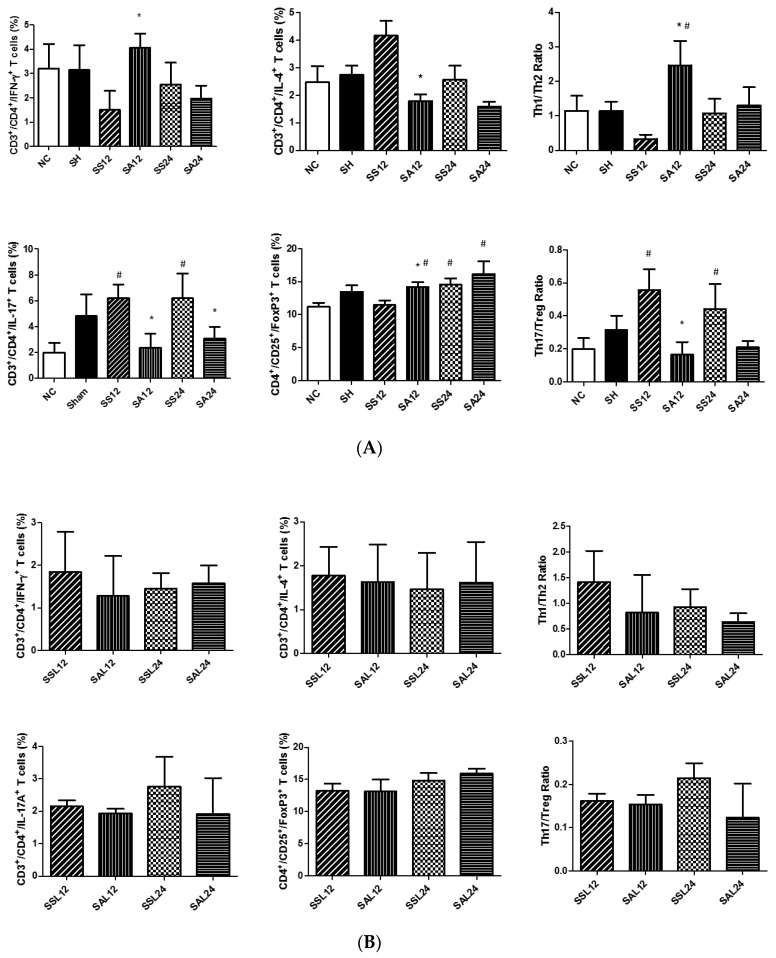
Percentages of (**A**) CD4+ T-cell subsets including Th1, Th2, Th17, regulatory T (Treg) cell distributions, and the Th1/Th2 and Th17/Treg ratios and (**B**) Th cell subsets and the Th1/Th2 and Th17/Treg ratios in para-aortic lymph nodes in sepsis groups with an inducible nitric oxide synthase (iNOS) inhibitor in para-aortic lymph nodes. Representatives of the Th cell phenotypes and groups are described in the legend of Figure 3. Data were analyzed using a one-way ANOVA with Tukey’s post hoc test and are presented as the mean ± SD. *n* = 8 for each group. ^#^ Significantly differs from the NC group (*p* < 0.05). * Significantly differs from the SS group at the same time point (*p* < 0.05).

Sepsis led to a decrement in CD4^+^ T-cell percentages in lymph nodes after CLP. The sepsis group with Arg administration maintained its CD4^+^ T-cell percentage and exhibited no differences from the NC or SH groups (Figure 5). Concerning subsets of CD4^+^ T cells, the changing pattern of subpopulation distributions was similar to that observed in the blood. No differences in Th1, Th2 percentages, and Th1/Th2 ratio were seen between the SS and NC groups. However, percentages of Th17 cells and the Th17/Treg ratio in the SS group were significantly higher than those in the NC group at both 12 and 24 h post-CLP. Compared to the SS group, the SA group exhibited higher Th1, Treg, and a Th1/Th2 ratio and lower Th2, Th17, and a Th17/Treg ratio at 12 and/or 24 h after CLP (Figure 6A). Alterations in the Th/Treg distribution observed in the sepsis Arg group were abrogated when L-NIL was administered. No differences in Th1/Th2/Th17/Treg percentages were noted between the SSL and SAL groups at both time points post-CLP (Figure 6B).

### 3.5. Gene Expression Levels in the Liver

**Figure 7:**
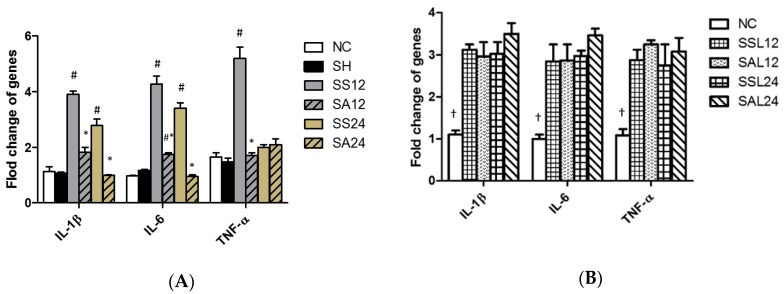
Expression of inflammatory mediator genes in the liver. (**A**) Interleukin (IL)-1β, IL-6, and tumor necrosis factor (TNF)-α mRNA expressions of the control and the experimental groups. NC, normal control group; SH, sham group; SS12, septic saline group sacrificed at 12 h after cecal ligation and puncture (CLP); SA12, septic arginine (Arg) group sacrificed at 12 h after CLP; SS24, septic saline group sacrificed at 24 h after CLP; SA24, septic Arg group sacrificed at 24 h after CLP. ^#^ Significantly differs from NC group (*p* < 0.05). * Significantly differs from the SS groups at the same time point (*p* < 0.05). (**B**) Sepsis groups with inducible nitric oxide (iNOS) inhibitor at 12 h and 24 h post-CLP. SSL12, SS12 group with iNOS inhibitor; SAL12, SA12 with the iNOS inhibitor; SSL24, SS24 with the iNOS inhibitor; SAL24, SA24 with the iNOS inhibitor. + Significantly differs from all the sepsis groups at 2 time points (*p* < 0.05). Messenger RNA changes were quantitated by qPCR and calculated using the comparative CT (2^−ΔΔCt^) method. mRNA expression levels in the normal control group were used as a calibrator. Data are shown as the mean ± SD. *n* = 8 for each group. Data were analyzed using a one-way ANOVA with Tukey’s post hoc test.

Compared to the NC and SH groups, the expression levels of IL-1β, IL-6, and TNF-α genes were higher in the SS group at 12 h or at both 12 and 24 h after CLP. The IL-1β and IL-6 gene expressions were significantly lower at both 12 and 24 h while TNF-α expression was lower at 12 h post-CLP in the SA group compared to the SS group (Figure 7A). However, no differences in gene expression levels were found between the two sepsis groups with L-NIL at either time point (Figure 7B).

## 4. Discussion

Arg is commonly present as one of the components in enteral formulas which are given to surgical or critically ill patients [31]. According to previous clinical studies, the impact of Arg-containing formulas differs from no effects on clinical outcomes [20,21] to significantly increasing mortality [22]. However, there is also a study which reported a significant reduction in infection and tendency toward reduced mortality [32]. The inconsistent results may have been confounded by different formulations, used dosages, administration routes, and populations of recruited patients. Studies which examined Arg monotherapy are rare. A clinical trial found that a continuous infusion of Arg enhanced Arg concentrations and NO production without adverse events in septic patients [23]. A recent report by Luiking et al. revealed that prolonged intravenous Arg administration increased plasma Arg and whole-body NO synthesis and was safe in terms of global hemodynamics in patients with septic shock [33]. In this study, we administered a single dose of intravenous Arg 1 h after CLP to account for the expected Arg decrement in the blood because a previous study showed that arterial concentrations of Arg dropped as early as 90 min in a lipopolysaccharide-induced sepsis rodent model [34]. Since Th/Treg lymphocytes from the blood and lymphoid organs play crucial roles in modulating homeostasis of immune responses during sepsis, we analyzed Th1, Th2, Th17, and Treg cell distributions by identifying cytokines produced from different CD4^+^ T-cell subsets in the blood and abdominal lymph nodes in this study. We found that Arg administration after sepsis increased the availability of Arg and produced more-balanced Th/Treg polarization that may have favorable effects on attenuating liver inflammation and injury. Inducible NO inhibition abrogates the beneficial effects of the Th cell distribution, suggesting that an Arg–NO pathway may partly contribute to modulating the homeostasis of Th/Treg lymphocytes during sepsis.

A previous study showed that sepsis leads to reductions in T lymphocyte subsets and impairment of T-cell functions [5]. A septic condition caused the host response to shift from a Th1 toward a Th2 phenotype after an episode of hyperinflammation [35]. Meanwhile, it was reported that plasma IL-17A derived from Th17 cells increased after CLP. IL-17A promotes excessive inflammatory cytokine production that may result in adverse outcomes in experimental sepsis [36]. A study by Gupta et al. found that the circulating Th17/Treg ratio was significantly higher in non-survivors compared to surviving septic patients. The balance of Th1/Th2 and Th17/Treg changing toward a Th2 and Th17 response may contribute to the progression of sepsis [7]. Findings of our study revealed that sepsis resulted in decreased CD4^+^ cell populations, lower Th1/Th2 ratios, and higher Th17/Treg ratios in the circulation and abdominal lymph nodes. These results were consistent with previous reports that sepsis results in T-cell depletion and dysregulation.

Arg is essential for the transcription and expression of the T-cell receptor, as well as regulating the cell cycle of T lymphocytes [37]. It was reported that Arg supplementation promoted the production of IFN-γ by Th1 cells in experimental cerebral malaria [38]. In this study, we found that Arg administration reversed the decrement in CD4^+^ lymphocytes and exerted more-balanced Th1/Th2 and Th17/Treg distributions either in circulation or lymph nodes near the infectious site. This may have attenuated sepsis-induced systemic and remote organ inflammation. The liver plays crucial roles in metabolic hemostasis and immunology. Liver dysfunction is an independent risk factor for multiorgan dysfunction after sepsis [4]. Liver injury was noted at 6 h and was distinct at 24 h after CLP [39]. We found that septic mice with an Arg injection had lower expressions of hepatic inflammatory mediators. Decreased plasma liver injury markers and galectin-3 levels were also observed. The galectin-3 protein is involved in the phagocytic process of apoptotic hepatocytes [40]. Galectin-3 is one of the most frequently identified markers of liver disease [41] and is considered a prognostic biomarker in severe trauma or sepsis patients [42,43,44]. These findings indicated that hepatic inflammation and injury were attenuated when Arg was administered after CLP. Previous studies indicated that an unbalanced Th1/Th2 response and overexpression of Th17 cells are characteristics of liver inflammation and damage [45]. Downregulation of the CD4^+^ T-cell lineage toward Th2 and Th17 may alleviate liver inflammation and subsequent liver damage during sepsis.

NO is essential in regulating various functions in sepsis. NO is a direct toxin to most bacteria and was found to have free radical-scavenging properties that may reduce organ dysfunction and injury [46]. One study showed that universal inhibition of NO synthesis worsens the outcomes [47]. However, exaggerated NO production may also have detrimental effects in sepsis and septic shock [48,49,50,51]. This may indicate that NO is a double-edged sword during sepsis. Arg is a dominant factor in regulating NO production during catabolic conditions [25]. Since arginase is upregulated and nNOS and eNOS are downregulated during sepsis, the availability of Arg and subsequent NO synthesis may be limited, and upregulation of iNOS may compensate for the reduced NO [15,26]. The findings of our study showed that the favorable effects observed in the Arg sepsis group were abrogated when L-NIL was administered. These findings suggest that NO is at least partly responsible for regulating the homeostasis of Th/Treg cells and subsequent organ inflammation during sepsis. However, the administration route and supplemented dosage are critical to the availability of Arg. A previous study demonstrated that intravenous Arg administered after initiation of sepsis improved survival compared to enteral feeding [52]. Sepsis may result in impaired absorptive gut function and intravenous administration may increase the effectiveness of Arg. In this study, the dosage of intravenous Arg used immediately after CLP may have increased Arg and NO availability, favored CD4^+^ T-cell homeostasis, and subsequently attenuated organ inflammation.

A previous study showed that there is a close association between Th1 cells and NO in diseases [53]. In an animal study, Niedbala et al. found that production of low doses of NO immediately after infection selectively enhanced murine Th1 cell differentiation but not Th2 [54]. Higher Th1/Th2 ratios in the blood and para-aortic lymph nodes observed in this study may possibly have resulted from NO derived from Arg administration after CLP. There may be other benefits of Arg-mediated mechanisms involved in mitigating organ injury. Exogenous Arg may compensate for the depletion of Arg that potentially reduces catabolism and promotes wound healing in catabolic conditions [16,55]. Otherwise, Arg supplementation may restore vascular NO formation and thus improve microcirculation and vascular endothelial function, thereby ameliorating organ injury [19,23,56]. Elucidating the exact mechanisms responsible for the influence of Arg on Th cell polarization and organ damage requires further investigation. On the other hand, supplementation with essential amino acids has been demonstrated to efficiently support immune function in humans [57]. An alternative infusion of an isonitrogenous essential amino acid mixture comparable to Arg may be worth testing in the future.

## 5. Conclusions

Arg administration increased Arg availability, maintained CD4^+^ T-cell percentages in the circulation and para-aortic lymph nodes, inhibited the polarization of blood CD4^+^ T cells toward Th2 and Th17 responses that may have favorable effects on restoring Th/Treg cell dysregulation, and alleviated liver inflammation and injury. Inducible NO derived from Arg may be partly involved in modulating the homeostasis of Th/Treg cells and subsequent liver inflammation during sepsis. The findings of this study provide basic information and imply that a single dose of Arg may produce more-balanced Th cell polarization and alleviate remote liver injury in surgical patients with high risk of abdominal infectious complications, which may benefit early recovery after surgery (ERAS).

## Figures and Tables

**Table 1:** Primers for quantitative real-time PCR.

Gene	Primer Sequence
IL-1β	F: 5′-TGCCACCTTTTGACAGTGATG-3′ R: 5′-ATGTGCTGCTGCGAGATTTG-3′
IL-6	F: 5′-TCCTACCCCAACTTCCAATGCTC-3′ R: 5′-TTGGATGGTCTTGGTCCTTAGCC-3′
TNF-α	F: 5′-ATGGCCTCCCTCTCATCAGT-3′ R: 5′-TTTGCTACGACGTGGGCTAC-3′
β-actin	F: 5′-GTCAGAAGGACTCCTATGTG-3′ R: 5′-ACGCAGCTCATTGTAGAAG-3′

Abbreviations: IL-1β, interleukin-1β; IL-6, interleukin-6; TNF-α, tumor necrosis factor-α; F, forward primer; R, reverse primer.

**Table 2:** Plasma biochemical indicators and liver malondialdehyde (MDA) levels.

**Parameters**	**NC**	**SS12**	**SA12**	**SS24**	**SA24**
Plasma					
AST (U/L)	58.2 ± 2.4	449.1 ± 35.3 ^#^	130.9 ± 7.7 ^#,^*	224.4 ± 16.6 ^#^	87.87 ± 1.93 ^#,^*
ALT (U/L)	37.3 ± 5.5	611.7 ± 71.9 ^#^	251.9 ± 6.1^#,^*	387.6 ± 39.4 ^#^	217.4 ± 25.38 ^#,^*
Gal-3 (ng/mL)	6.3 ± 0.9	25.9 ± 3.2 ^#^	11.1 ± 1.4 ^#,^*	20.2 ± 3.1^#^	9.2 ± 1.2 *
Liver					
MDA (nmol/mg protein)	3.26 ± 0.6	6.5 ± 0.3 ^#^	4.8 ± 0.4 *	5.7 ± 0.3 ^#^	3.8 ± 0.2 *
**Parameters**	**SH**	**SSL12**	**SAL12**	**SSL24**	**SAL24**
Plasma					
AST (U/L)	60.5 ± 3.1	368.8 ± 40.5 ^#^	346.5 ± 38.2 ^#^	222.5 ± 46.3 ^#^	216.5 ± 35.6 ^#^
ALT (U/L)	40.5 ± 5.6	564.3 ± 50.6 ^#^	462.3 ± 41.3 ^#^	326.5 ± 35.6 ^#^	200.4 ± 43.5 ^#^
Gal-3 (ng/mL)	7.1 ± 0.6	24.5 ± 1.3 ^#^	22.6 ± 1.1 ^#^	23.6 ± 1.3 ^#^	20.5 ± 1.2 ^#^
Liver					
MDA (nmol/mg protein)	4.1 ± 0.5	7.5 ± 0.2 ^#^	6.6 ± 0.3 ^#^	7.3 ± 0.5 ^#^	7.1 ± 0.2 ^#^

Data are presented as the mean ± SD. NC, normal control group; SH, sham group; SS12, septic saline group sacrificed at 12 h after cecal ligation and puncture (CLP); SA12, septic arginine (Arg) group sacrificed at 12 h after CLP; SS24, septic saline group sacrificed at 24 h after CLP; SA24, septic Arg group sacrificed at 24 h after CLP. SSL12, SS12 group with an inducible nitric oxide synthase (iNOS) inhibitor; SAL12, SA12 with the iNOS inhibitor; SSL24, SS24 with the iNOS inhibitor; SAL24, SA24 with the iNOS inhibitor. Abbreviations: ALT, alanine transaminase; AST, aspartate transaminase; Gal-3, galactin-3. All data are representative of duplicate measurements (*n* = 8). Differences among groups were analyzed by a one-way analysis of variance (ANOVA) followed by Tukey’s post hoc test. ^#^ Significantly differs from the NC group (*p* < 0.05). * Significantly differs from the SS group at the same time point (*p* < 0.05).
